# Strategies for Prevention and Control of Vibriosis in Asian Fish Culture

**DOI:** 10.3390/vaccines11010098

**Published:** 2022-12-31

**Authors:** Kangping Xu, Yushu Wang, Wangxiaohan Yang, Hongyan Cai, Youyu Zhang, Lixing Huang

**Affiliations:** 1 Fisheries College, Key Laboratory of Healthy Mariculture for the East China Sea, Ministry of Agriculture, Jimei University, Xiamen 361021, China; 2 Institute of Electromagnetics and Acoustics, School of Electronic Science and Engineering, Xiamen University, Xiamen 361005, China; 3 Fisheries College, Fujian Engineering Research Center of Aquatic Breeding and Healthy Aquaculture, Jimei University, Xiamen 361021, China

**Keywords:** Asian, fish culture, vibriosis, prevention, control strategies, vaccines

## Abstract

It is estimated that vibriosis account for about half of the economic losses in Asian fish culture. Consequently, the prevention and control of vibriosis is one of the priority research topics in the field of Asian fish culture disease. Relevant measures have been proposed to control some *Vibrios* that pose a threat to Asian fish culture, but there are currently only a few effective vaccines available to combat these *Vibrios*. The purpose of our review is to sum up the main prevention methods and the latest control strategies of seven *Vibrio* species that cause great harm to Asian aquaculture, including *Vibrio harveyi*, *Vibrio vulnificus*, *Vibrio parahaemolyticus*, *Vibrio mimicus*, *Vibrio anguillarum*, *Vibrio alginolyticus* and *Vibrio cholerae*. Strategies such as antibiotics, probiotics, bacteriophages, antimicrobials from plants and other natural sources, as well as vaccines, are compared and discussed here. We expect this review will provide some new views and recommendations for the future better prevention and control of vibriosis in Asian fish culture.

## 1. Introduction

Twenty years ago, aquatic products played a secondary role in people’s food choices. However, now aquatic products have become one of the mainstream food categories. Looking back on the development of global aquaculture from 1997 to 2017, aquaculture has made a substantial contribution to food production throughout the world, especially in Asia. According to the current consumption, aquaculture production needs to increase from 82,087 kilotons in 2018 to 129,000 kilotons in 2050 to meet global needs [1,2]. By 2050, aquaculture will dominate the global seafood supply [3].

*Vibrio* is one of the important pathogenic microorganisms of humans and marine animals. It widely exists in marine and freshwater ecosystems. Because of its high abundance and biomass, *Vibrio* plays a crucial role in the aquatic environment. More than 80 species of *Vibrio* have been reported, some of which are pathogenic to animals, especially aquatic animals, some to humans, and some to both animals and humans [4]. The outbreak of vibriosis will not only seriously affect marine biomass but also lead to serious economic losses in Asian fish culture.

With the rapid development of Asian fish culture in recent decades, the cases of *Vibrio* infection through aquatic products at home and abroad, causing human disease or huge economic losses, are also increasing year by year. At the same time, the prevention and control measures for *Vibrio* are also developing. At present, the use of antibiotics is the most important treatment for vibriosis in Asian fish culture [5]. At the same time, the overuse of broad-spectrum antibiotics has resulted in an increase in the number of drug-resistant bacteria. The resistance genes of these bacteria can be transferred to other bacteria that have never been exposed to the antibiotic [6]. Therefore, it is necessary to develop some antibiotic-free methods. For example, using vaccines, probiotics, bacteriophages and other technologies.

Before considering the prevention and control of *Vibrio*, it is essential first to identify the exact pathogen. At present, the mainly used identification methods are still conventional physiological, biochemical analyses, 16S rDNA sequencing and drug sensitivity test. In addition to these widely used assays, some convenient, fast and highly sensitive detection methods have been developed in recent years, for example, the identification of biomarkers based on host genes [7], exosomic miRNAs [8] and so on.

Vaccination in Asian fish culture can prevent or mitigate the spread of disease and is effective against many related pathogens [9]. Vaccination is usually a secure and economic precaution. For this reason, illness prevention based on stimulating the immune system of aquatic animals has proved to be the basis of the development of modern Asian fish culture. Nevertheless, there are only a few *Vibrios* with vaccine control technology.

**Figure 1:**
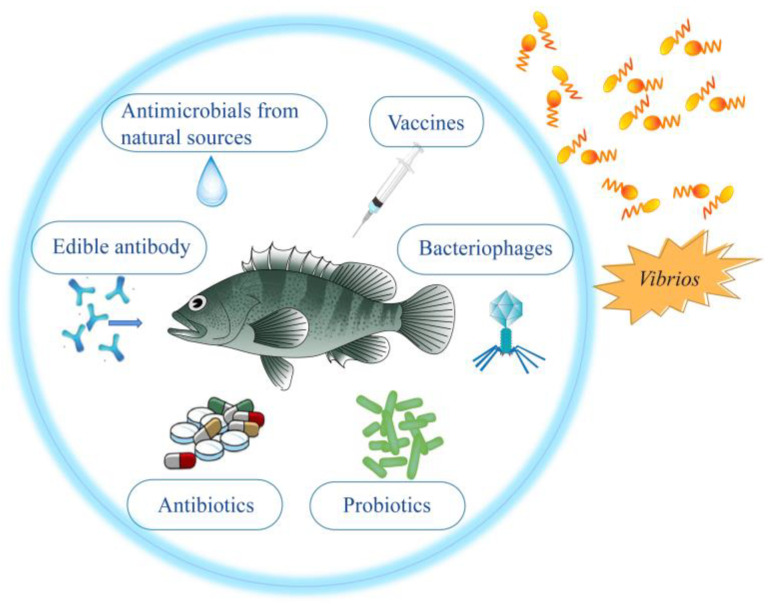
Strategies for prevention and control of vibriosis in Asian fish culture mentioned in this review.

This review discusses the control and prevention strategies of seven *Vibrio* species that are seriously harmful to Asian fish culture, including *Vibrio harveyi*, *Vibrio vulnificus*, *Vibrio parahaemolyticus*, *Vibrio mimicus*, *Vibrio anguillarum*, *Vibrio alginolyticus* and *Vibrio cholerae*. For each *Vibrio*, we describe their prevention and treatment methods (Figure 1), especially vaccine prevention methods, in order to provide views for better prevention and control of vibriosis in Asian fish culture in the future.

## 2. Control and Prevention Strategies of *Vibrios*

### 2.1. V. harveyi

*V. harveyi* is a luminous marine bacterium and is also a well-recognized and acute pathogen of marine fish [10]. The research on the control and prevention measures of *V. harveyi* started early, and now there are a variety of control technologies (Table 1).

#### 2.1.1. Antibiotics

Antibiotic methods are generally used in the initial stage of prevention and treatment of vibriosis or emergency treatment. In the case of skin ulcer disease of young hybrid groupers, researchers confirm that the pathogen of this sickness is *V. harveyi* ML01 strain, which is sensitive to minocycline, doxycycline and ceftriaxone. In other words, these three antibiotics can be used for emergency treatment of *V. harveyi* infection [11]. In 2017, the drug sensitivity test of *V. harveyi* extracted from the diseased cultured hippocampus was carried out, and the results showed that *V. harveyi* is highly sensitive to doxycycline and tetracycline. This provides a reference for the prevention strategy of vibriosis in seahorse culture in eastern China [12]. Although antibiotics are widely used, the rapid increase in antibiotic resistance is really puzzling.

#### 2.1.2. Bacteriophages

As people gradually realize the risk of using antibiotics in Asian fish culture, probiotics, bacteriophage, antimicrobials from natural sources and so on are gradually replacing antibiotics. In bacteriophage therapy, phages such as lytic *Vibrio* phage VhKM4 [13] can resist *V. harveyi* efficiently due to its strong lytic activity. Although several research studies have proven these methods effective, there have not been enough similar studies of each method to prove that they should be promoted in practical application. At the same time, whether these biological control methods have potential threats still needs further study in future research.

#### 2.1.3. Vaccines

One of the research hot spots of *Vibrio* prevention is vaccine development. Most research on *V. harveyi* vaccine is targeted at fish. Many excellent achievements have been made in the research of *V. harveyi* vaccine. Vaccine exploration started from the traditional whole-cell inactivation method, followed by the study on the method of purifying subcellular components, making vaccine technology enter the era of modern vaccines represented by DNA vaccine [10].

Whole-cell vaccines can be categorized into two types, attenuated live vaccine and inactivated vaccine. The production cost of these vaccines is not high [14]. This traditional vaccine is the most widely used in the prevention of aquatic animal diseases.

- Inactivated vaccines

FKVh, a vaccine mainly composed of formalin-inactivated *V. harveyi* Vh1 strain, may be an effective vaccine, and the survival rate of hybrid tilapia increased from 20% to 87% after vaccination [15]. There is also a combined vaccine (VICV) against *V. vulnificus*, *V. alginolyticus*, *V. harveyi* and infectious spleen and kidney necrosis virus (ISKNV). Huang et al. have proved its immunization effectiveness by immunizing orange-spotted grouper *Epinephelus coioides* with the VICV vaccine and attacking the above four pathogens [16]. Compared with the monovalent vaccine, this kind of vaccine can more conveniently protect fish from a variety of pathogens.

Although the production cost of inactivated vaccines is relatively lower compared with other kinds of vaccines, their performance still needs to be continuously improved. This can be achieved by combining adjuvants, liposome embedding and other methods. Research has proposed a greatly effective vaccine that can prevent *V. harveyi*. The vaccine consists of inactivated *V. harveyi* cells and ISA763 AVG adjuvant. The experiment observed that the RPS of grouper inoculated with this vaccine was 100% in the sixth week and 91.7% in the twelfth week after being attacked by *V. harveyi* [17]. The formalin-inactivated cell of *V. harveyi* adjuvanted with Montanide TMISA 763 AVG induced efficient immune protection in turbot [18]. Similarly, the application of liposomes-entrapped *V. harveyi* WCV or *V. harveyi* WC can actively strengthen the immune system and provide protection for *V. harveyi* infection in *Epinephelus bruneus* [19]. The expression standard of various immune substances in the grouper‘s spleen is significantly up-regulated after inoculation in the laboratory, using a vaccine made of inactivated *V. harveyi* ZJ0603 combined with β-glucan [20].

- Attenuated live vaccines

An attenuated live vaccine has been developed to highly protect Japanese flounder (*Paralichthys olivaceus*) infected with *V. harveyi* in the experiment. The vaccine is made of live *Escherichia coli*, which can express and secrete Vhp1 with impaired cytotoxicity [21]. Moreover, a study shows that *V. harveyi* WC13DH51 strain can be made into a live attenuated vaccine and has a significant protective effect on groupers [22]. Furthermore, an attenuated live vaccine was developed by constructing recombinant Et15VhD. The infection experiment shows that this vaccine can effectively prevent the infection of *V. harveyi* and *E. tarda* [23]. Similarly, the attenuated mutant strain T4DM of *V. harveyi* can also be used as a live attenuated vaccine. On the medium containing rifampicin with increased concentration, T4DM was obtained by selecting T4D mutants repeatedly with a relatively narrow antibiotic resistance profile and no detectable plasmid. T4DM is also a cross-protection vaccine, which can effectively protect Japanese flounder from the infection of *V. alginolyticusvia* and *V. harveyi*, especially through immersion (10^8^ CFU/mL) and intraperitoneal injection (10^8^ CFU/mL) [24].

- Subunit vaccines

A subunit vaccine made of purified recombinant Vhp1 can effectively render Japanese flounder *V. harveyi*-resistant [21]. There is also a *V. harveyi* subunit vaccine encoding TssJ antigen that was found to emerge a moderate protective role against *V. harveyi* in fish. The full-length sequence of TssJ was obtained from the *V. harveyi* strain QT520 and was predicted as a new candidate antigen, whose relative percentage survival was 52.39% [25]. Moreover, based on VirB11, a recombinant protein vaccine was developed and became a candidate vaccine to prevent *V. harveyi* infection [26]. In addition, recombinant cell vaccines expressing the DnaJ and OmpK have strong cross-protection against *V. alginolyticus, V. parahaemolyticus* and *V. harveyi* [27].

- Anti-idiotypic vaccines

A great deal of studies have shown that antibodies may have a regulatory effect on the immune system. Consequently, they have the conditions for making vaccines. The vaccine developed according to this principle is called an anti-idiotypic vaccine. As an anti-Id vaccine, anti-Id IgG is a vaccine that can provide protection by imitating the antigen epitope of *V. harveyi*. It may have a good application prospect in Asian fish culture against *V. harveyi* [28].

- DNA vaccines

A series of experimental results suggest that DNA vaccines represented by pDV are positive vaccines against *V. harveyi* [29]. DNA vaccine can also be obtained by cloning the *ompU* gene into pEGFP-N1 plasmid. After the infection test of the turbot, the RPS was 51.4% [30]. Moreover, a *V. harveyi* DNA vaccine encoding TssJ antigen could produce a moderate protective role against *V. harveyi* in fish, and the relative percentage survival was 69.11% [25]. However, the exact route of protection in fish for these vaccines is still unclear at present [14].

- mRNA vaccines

In 1990, the successful use of in vitro transcription (IVT) mRNA in animals was first reported, and related research has developed extremely rapidly since then. The production cost of the mRNA vaccine is low. The application safety is high, and the development turnaround time is short and pretty efficient. Therefore, the mRNA vaccine may have a better prospect compared with the traditional vaccine [31]. mRNA vaccines applied to aquatic animals are rare. We found a study on the mRNA vaccine against *V. harveyi* infection in fish. In this study, the researchers first used computational techniques to find potential T-and B-cell epitopes in *V. harveyi* hemolysin proteins and then sutured these epitopes into multi epitope mRNA vaccines. However, more experiments are needed to further prove the effectiveness of this vaccine [32].

**Table 1:** Control and prevention strategies of *V. harveyi.* NR: Not Relevant, None: the method has not been tested in vivo or relevant data has not been found.

Pathogen	Prevention and Control Technology	Concrete Measure/ Vaccine Type	Host	Vaccine Antigen Components	Route of Infection	Ref.
*V. harveyi*	Antibiotics	Ceftriaxone, Doxycycline, Minocycline	Juvenile hybrid groupers	NR	Bath, Injection (IP)	[11]
		Doxycycline, Tetracycline	Hippocampus	NR	Injection (IP)	[12]
	Bacteriophages	Phage VhKM4	Finfish	NR		[13]
	Vaccines	Inactivated	Marine Red Hybrid Tilapia	*V. harveyi* strain Vh1 (Formalin-Inactivated)	Injection (IP)	[15]
			*E. coioides*	VICV	Injection (IP)	[16]
			Orange-spotted grouper	*V. Harveyi* (formalin-killed, Adjuvant: ISA763 AVG)	Injection (IP)	[17]
			Turbot	*V. Harveyi* (formalin-killed, Adjuvant: TMISA763 AVG)	Injection (IP)	[18]
			Pearl gentian grouper	*V. harveyi* ZJ0603 (Formalin-killed, combine with β-glucanhas)	Injection (IP)	[20]
		Attenuated	Grouper	Non-toxic *V. harveyi*	Bath, Injection (IP)	[22]
			Japanese flounder	Attenuated mutant *V. Harveyi* T4DM	Bath, Injection (IP)	[24]
		Subunit	Japanese flounder	Recombinant Vhp1	Injection (IP)	[21]
			Golden pompano	Antigen encoding TssJ	Injection (IP)	[25]
			Orange-spotted grouper	VirB11	Injection (IP)	[26]
			Juvenile sea bass	Expressed r-OmpK of *Vibrio*	Injection (IP)	[27]
		Anti-idiotypic	Grouper	Anti-Id IgG (Fab)	Injection (IP)	[28]
		DNA	Japanese flounder	Plasmid pDV	Injection (IP, IM)	[29]
			Turbot	Plasmid with OmpU	Injection (IP, IM)	[30]
		mRNA	Fish	and B-cell epitopes in hemolysin protein	None	[32]

### 2.2. V. vulnificus

*V. vulnificus* is a gram-negative bacterium that can cause wound infection and septicemia. Unlike other *Vibrios*, it is able to ferment lactose. According to genetic, biochemical and serological tests and host infection, *V. vulnificus* is currently classified into three biotypes. Biotype 1 strains are the source of most human infections, and biotype 2 strains mainly infect eels. The recently discovered biotype 3 has the biochemical characteristics of biotype 2 and 1 and can result in human wound infection [33].

In this era of environmental protection and sustainable development, the biological control strategy of *Vibrio* is gradually emerging, but there are few examples in fish farming.

#### Vaccines

- Inactivated vaccines

Since *V. vulnificus* is one of the most harmful *Vibrios* to Asian fish culture, its vaccine control technology has been continuously developed (Table 2). The early vaccines against *V. vulnificus* are generally inactivated vaccines. For example, a formalin-inactivated *V. vulnificus* vaccine was effective at exciting a humoral antibody response in sex-reversed hybrid tilapia [34]. The combined vaccine (VICV) also can be effective against *V. harveyi* [16].

- Subunit vaccines

There are also a few subunit vaccines against *V. vulnificus* under development. Some scientists have found that the expressed OmpU of *V. vulnificus* was capable of resisting the infection of *V. vulnificus* in Japanese eels and evidently raised the immune ability of eel. Therefore, the OmpU is proposed as a potential subunit vaccine against *V. vulnificus* [35].

- Multivalent vaccines

Multivalent vaccines may be more practical in aquaculture due to their multiple protective effects. There is a bivalent protein that can be used against *V. vulnificus* and *Edwardsiella anguillarum* in Japanese eel as a vaccine. This fresh recombinant Omp vaccine with OmpA and OmpU shows its strong immunogenicity by significantly increasing the RPS rate of eels when infected with *E. anguillarum* and *V. vulnificus* [36].

In addition to these bivalent vaccines, a trivalent outer membrane protein, OmpII-U-A, containing part sequences of OmpU from *V. vulnificus*, OmpA from *E. anguillarum,* and OmpII from *A. hydrophila*, can also be made into a vaccine. According to the study of He et al. [37], the OmpII-U-A is able to prevent eel from being infected by *V. vulnificus* and *A. hydrophila*. This is the first time the expression and immunogenicity of a trivalent Omp are being reported, and the outcomes of this research will supply valuable guidelines for the exploration of multiplex vaccines in fish.

*V. vulnificus* has many bivalent and trivalent vaccines that can protect aquatic animals from *A. hydrophila*, *E. anguillarum* and other pathogens. These vaccines can provide ideas for the advancement of aquatic animal multiplex vaccines.

### 2.3. V. parahaemolyticus

*V. parahaemolyticus* is a Gram-negative, slightly halophilic bacterium that inhabits brackish aquatic environments such as coastal and estuarine waters. Apart from being pathogenic to aquatic organisms, *V. parahaemolyticus* is also known as a global food-borne pathogen and one of the most common causes of gastroenteritis in East Asia due to the local dietary habit of eating raw fish and shellfish [38,39].

*V. parahaemolyticus* is antibiotic-resistant, so it cannot be treated with antibiotics which is currently the most commonly used measure in Asian fish culture [40]. Consequently, there is a pressing need to exploit fresh, effective alternatives for antibiotics against *V. parahaemolyticus* (Table 3), while the vaccine is the most promising approach due to its economy, efficacy and safety in public awareness [40,41,42].

#### Vaccines

- Inactivated vaccines

A polyvalent *Vibrio* vaccine had already been commercially used in Indonesian fish farming recently for tiger grouper (*Mycteroperca tigris*) and had shown effective protection against *V. parahaemolyticus* and two other *Vibrio* pathogens [43].

- Recombinant vaccines

A study on cross-protection also found a recombinant cell vaccine had successfully induced an immune response to *V. parahaemolyticus* in juvenile sea bass by expressing the OmpK of *Vibrio* [27]. At the same time, another study has pointed out the limitation of recombinant OmpK in preparing diagnostic antibodies [44]. For this reason, using modern methods for understanding and developing new *V. parahaemolyticus* immunogenic proteins and antibodies are necessary [45,46].

### 2.4. V. cholerae

*V. cholerae* is a Gram-negative motile bacterium that can cause fatal pandemic diseases. There are millions of cholera cases worldwide every year, and the mortality rate is extremely high [47]. Consuming contaminated seafood by mistake is one of the reasons why people are infected with *V. cholerae*. As an important food-borne pathogen, *V. cholerae* is widely distributed in fish, which brings serious safety hazards to human and aquatic animal health [48].

#### 2.4.1. Antibiotics

*V. cholerae* is one of the important pathogens related to fish vibriosis. In the bluegill sunfish that died in the farms of Guangdong around 2018, the pathogen identified was non-O1/non-O139 *V. cholerae.* The antibiotic sensitivity displayed that the isolated strain was sensitive to azithromycin, chloramphenicol, neomycin, norfloxacin, doxycycline, etc. The possible method to prevent infection of bluegill sunfish is to give neomycin or doxycycline for seven days [49].

#### 2.4.2. Vaccines

After consulting a large number of data, we found that the current prevention and control of *V. cholerae* in Asian fish culture is still based on antibiotics, and no *V. cholerae* vaccine for aquatic animals has been found yet. Nevertheless, in recent decades, the misuse of antibiotics has resulted in the emergence and spread of drug-resistant bacteria in the environment, which is likely to pose a threat to public health [50]. Therefore, people are also constantly exploring new methods for the prevention and control of *V. cholerae* (Table 4).

#### 2.4.3. Edible Antibodies

In 2015, a dominant non-O1 *V. cholerae* L1 strain was isolated from diseased carp in a breeding farm in Jiangsu, China. The researchers used egg yolk powder (IgY) against non-O1 vibrio cholerae to prove its effective effect on diseased carp [51]. This is one of the new methods to control *V. cholerae*.

#### 2.4.4. Bacteriophages

In addition to the above emerging strategies, a phage prevention and control method has also been proposed [52], which has become a highly potential prevention and control measure in the future. Nevertheless, we have not found any information about the bacteriophage therapy of *V. cholerae.* This may be due to the difficulty in developing efficient phage administration mechanisms, different types of aquaculture systems, and the lack of a specific regulatory frame [53].

As a kind of pathogenic bacteria that is very harmful to Asian fish culture, the lack of *V. cholerae* vaccine prevention technology is indeed a big gap in the prevention of aquatic animal diseases.

### 2.5. V. anguillarum

*V. anguillarum* is a Gram-negative, comma-shaped rod bacterium that is polarly flagellated, non-sporeforming, halophilic and facultatively anaerobic. It is pathogenic to both marine and freshwater living animals, especially fish, showing classic symptoms of vibriosis, such as lethargy, abdominal distension, skin lesion and even internal and external ulceration [54,55].

In most circumstances, infection occurs by penetrating fish skin as *V. anguillarum* can enter through injuries or damaged mucous layers easily, and oral ingestion of *V. anguillarum* would also lead to vibriosis occasionally [56]. According to some studies, the outbreak of *V. anguillarum* is triggered by several factors, including physical, chemical and biological stresses [54].

The prevalence of *V. anguillarum* virulence has increased gradually as a result of the expansion of aquaculture [54]. Moreover, Asian fish culture losses caused by *V. anguillarum*-induced mortalities are extremely overwhelming over the world today. Therefore, even if the prevention and treatment of *V. anguillarum* still face major challenges, it is always a high priority for international aquatic research.

Control of *V. anguillarum* is usually carried out through the application of water quality management, vaccines, antibiotics and probiotics [43]. The other preventative and treatment procedures may not be as effective as vaccination in high-quality aquaculture but are still indispensable in maintaining fish production and economic status.

#### 2.5.1. Antibiotics

The most common measure dealing with *V. anguillarum* infection in intensive Asian fish culture currently is still the use of chemicals and antibiotics [57], such as tetracycline, sulfaisozole and sulphamonomethioxine [55]. However, the intensive use of antibiotics leads to the bacterial resistance issue. That is precisely why scientists have made great efforts to find other effective and safe methods to control and prevent *V. anguillarum* infection. A successful example in Norwegian salmon farming shows that even with little use of antibiotics, fish production could still increase enormously [14].

In order to minimize the widespread utilization of antibiotics and to build a more sustainable Asian fish culture industry, scientists have investigated numerous alternatives to control *V. anguillarum,* including but not limited to antimicrobial peptides, probiotic bacterial strains, feed additives, immunostimulants and vaccines (Table 5).

#### 2.5.2. Vaccines

- Inactivated vaccines

Commercial aquaculture vaccines in the form of bacteria have mainly been used for the prevention of vibriosis for around 15 years in some countries and have played an important role in aquaculture [58]. These success cases encouraged and promoted the continuous study of inactivated vaccines to a great extent, and the inactivated vaccine is still the most common type of commercial vaccine against *V. anguillarum* until now. For example, Norvax-*Vibrio* Marine, AquaVac-*Vibrio,* ALPHA MARINE-*Vibrio* and MICRO ViB, which all composed of inactivated strains of both *V. anguillarum* serotypes O1 and O2 [54].

- Attenuated live vaccines

The genetically engineered live *V. anguillarum* vaccine for turbot (strain MVAV6203) obtained a national first-class new veterinary drug certificate in China in 2019, which is able to control the breeding mortality of turbot within 10% when combined with a live attenuated vaccine against *E. piscicida* [59]. This vaccine could protect turbots, oliver flounders and zebrafish efficiently either by immersion or injection administration in previous research [60] and has been proven to be suitable for Tiger puffer as well [61]. In some subsequent studies, the biosafety of the live attenuated vaccine MVAV6203 was significantly improved without affecting its immune protection efficacy with a controllable bacterial lysis system being converted to a live attenuated vaccine strain MVAV6203 of *V. anguillarum* [62].

- DNA and subunit vaccines

In addition to the use of whole-cell vaccines, we noticed that the development of DNA and subunit vaccines has been speeding up in recent years. Research and development of these two types of vaccines are mainly based on the immunological properties of *V. anguilluram* outer membrane proteins (OmpK, OmpU, OmpR, VAA) and flagellins (FlaA, FlaB) [63,64]. It was reported that a DNA vaccine based on the zinc metalloprotease EmpA already displayed gratifying results against *V. anguillarum* early in 2009 [65]. In recent years, several DNA and subunit vaccines based on *V. anguillarum* outer membrane proteins and their recombinant proteins have shown effective and long-lasting immunity [66,67,68,69]. It has also been proven in the study that particularly high protection against *V. anguillarum* infection exists when related proteins are formulated as bivalent vaccines [68].

Generally, injectable vaccines have the best protective effect on vibriosis, and intraperitoneal injection has been proven to be the most effective method of fish immunization [14]. In IP injection, oil adjuvants are usually used for protection. Nowadays, the preponderant vaccines on the market are polyvalent oil-adjuvanted vaccines. On the one hand, oil adjuvants have been related to side effects such as pigmentation, inflammation, growth disorders, visceral fiber adhesion and granulomatous lesion formation [70]. On the other hand, inactivated vaccines cannot provide ample protection unless adding adjuvants to enhance the effectiveness of the vaccine [14]. Thus, researchers have been looking for alternative adjuvants with fewer side effects and effective as well.

Adjuvants administrated with inactivated *V. anguillarum* vaccines are supposed to induce effective protection, similarly to other adjuvants, but cause fewer side effects [71,72]. It is the same case in autogenous vaccines, which confer high levels of immune protection and persistent immunity, but with high side effects. Thus, in order to increase protection and reduce or eliminate the side effects at the same time, liquid paraffin adjuvants have been suggested to be put into use [73]. Moreover, when it comes to DNA and subunit vaccines, adjuvants are mostly used to enhance the immune response of the vaccines [74,75].

The most non-negligible defect of injectable vaccines is that they are not suitable for either small or juvenile fish, which could easily cause secondary infections by injection. For this reason, even though not as effective as injectable vaccines, oral and immersion vaccines are still used in some areas of aquaculture. These two types of vaccines are based on *V. anguillarum* outer membrane proteins [76], *V. anguillarum* serotypes O1 and O2 [77], avirulent environmental isolates [78] or an attenuated *V. anguillarum* strain [79]. A conclusion can be made that the best answer for *V. angullarum* vaccine would be: easily manipulated, suitable for both adult and juvenile fish, and demonstrate effectiveness against a broad spectrum of *Vibrio* pathogens.

To create the ideal novel vaccine, undoubtedly, the most significant part will be the identification of common antigens [45]. The use of modern reverse vaccinology could make it come true by predicting potential protective antigens and epitopes, which could aid in narrowing down peptide selection in designing suitable vaccines [80].

**Table 5:** Control and prevention strategies of *V. anguillarum.* NR: Not Relevant, None: the method has not been tested in vivo or relevant data has not been found.

Pathogen	Prevention and Control Technology	Concrete Measure/ Vaccine Type	Host	Vaccine Antigen Components	Route of Infection	Ref.
*V. anguillarum*	Vaccines	Inactivated	Fish	Inactivated strains of *V. anguillarum* serotypes O2 and O1	Injection (IP)	[54]
		Attenuated	Turbot, Oliver flounder, Zebrafish, Tiger puffer	Genetically engineered live *V. anguillarum*	Injection	[59,60,61]
			Zebrafish	live attenuated *V. anguillarum* strain MVAV6203	Injection (IP)	[62]
		Subunit	*Paralichthys olivaceus*	Recombinant protein (rOmpK)	Injection	[69]
		DNA	Japanese flounder	Zinc metalloprotease EmpA	Injection (IM)	[65]
			*Paralichthys olivaceus*	Bicistronic plasmids (p-OmpK-CCL19 and p-OmpK-CCL4)	Injection (IM)	[66]
			Flounder	DNA plasmid encoding the VAA of *V. anguillarum*	Injection (IP)	[67]
			*Paralichthys olivaceus*	Plasmid *OmpK* from *V. anguillarum* plus cyclosporine A	Injection	[69]

### 2.6. V. mimicus

*V. mimicus* is a Gram-negative bacterium that is a species closely related to *V. cholerae*, and it is found to be distributed in freshwater, brackish water and seawater [81]. *V. mimicus* infection has a short disease course and high mortality, which has brought serious economic losses to Asian fish culture.

#### 2.6.1. Antibiotics

In 2011, *Vibrio mimicus* infection occurred in cultured *Pelteobagrus fulvidraco* in Guangdong and Guangxi in southern China [82]. Shortly afterward, a study published in 2014 showed that *V. mimicus* can also naturally infect southern catfish and Zhengchuan catfish and cause a large number of deaths. In the drug sensitivity test, all of the *V. mimicus* isolates from catfish were sensitive to gentamicin, florfenicol, lomefloxacin, and ciprofloxacin [83]. This provides ideas for the antibiotic control of *V. mimicus*.

#### 2.6.2. Vaccines

- DNA vaccines

In order to better prevent *V. mimicus* infection, the development of related excellent vaccines is also essential (Table 6). Since *V. mimicus* typically infects intestinal tracts, the idea of developing an oral DNA vaccine that can induce intestinal mucosal immunity is quite wise. Whereas, due to digestive tract degradation, oral naked DNA vaccine often has poor immunogenicity. Thereby, researchers considered using targeted DNA delivery strategies to improve the efficacy of vaccines. In recent years, based on the identification of the epitopes of OmpU and VMH 18 proteins from *V. mimicus,* a novel *V. mimicus* dual-targeted DNA vaccine constructed using BGS and ICLP as exogenous and endogenous targeted delivery vectors, respectively, was developed. Through the evaluation of the efficacy of oral administration to grass carp, it can be reliably concluded that this double-targeted DNA vaccine can cause remarkably higher systemic and intestinal mucosal immune protection than both single-targeted and naked DNA vaccines [84,85,86]. In its follow-up experiments, researchers have conducted some research on the impact of the vaccine on the response of grass carp intestinal microflora to the vaccine and the potential regulatory molecular mechanism of enhancing intestinal mucosal immunity [87,88].

- Attenuated live vaccines

In addition to DNA vaccines, the development of live attenuated vaccines against *V. mimicus* has also made some progress. Identifications of several possible virulence genes have been made in order to determine the complete genome sequence of *V. mimicus* strain sccf01 related to yellow catfish infection, which contributed to the development of subunit and lives attenuated vaccines by providing a genetic basis. Moreover, the immersion challenge test depicted that strain SCCF01 can adhere and colonize the mucosal surface, which is hopeful for the development of an attenuated vaccine [89].

The safety of attenuated vaccines can be effectively improved by introducing more than one attenuating phenotype. A successful example using the natural transformation of strain sccf01 to knock out genes provides a brand new way to construct targeted mutants in *V. mimicus* and also helps to eliminate the risk of virulence reversal caused by recombination events. By this means, the research on the pathogenic mechanism of *V. mimicus* and the development of attenuated vaccines have all been greatly improved [90].

### 2.7. V. alginolyticus

*V. alginolyticus* is a gram-negative bacterium that is usually distributed in coastal and estuarine environments and causes vibriosis in humans and fish. It has generated immense economic losses to the Asian fish culture industry and caused a menace to public hygiene [91].

#### 2.7.1. Antibiotics

Antibiotics are the most commonly used method to control *V. alginolyticus*. The drug sensitivity test of *V. alginolyticus* infected tilapia showed that treatment with florfenicol, enrofloxacin or terramycin could reduce the mortality of infected Nile tilapia [92]. What is more, the results of another drug sensitivity test on *V. alginolyticus* identified in the hippocampus cultured in eastern China showed that it was highly sensitive to doxycycline and tetracycline as well [12].

Even though *V. alginolyticus* remains sensitive to some antibiotics, with the widespread use of antibiotics, many studies have proved that *V. alginolyticus* has developed resistance to many antibiotics [93,94]; as a result, there is an urgent need to find some non-chemical control methods that can replace antibiotics.

#### 2.7.2. Probiotics

Probiotics have also been considered effective methods for *V. alginolyticus* controlling. For example, *Pseudoalteromonas* sp. IBRL PD4.8 is a promising natural antifouling agent that inhibits the growth of five contaminating bacteria and *V. alginolyticus* FB3 biofilms [95].

#### 2.7.3. Bacteriophages

A study revealed that with its extremely strong lytic effect, phage VP01 shows a strong underlying influence on the growth of *V. alginolyticus* and on biofilm formation [96]. Furthermore, it has been reported that a few bacteriophages may be powerful candidates for the treatment of *V. alginolyticus* infection, including bacteriophage VEN [97], HH109 [98], as well as phage valsw3-3 with stronger infectivity, better pH value and thermal stability [99]. Therefore, phage therapy tends to be a promising alternative approach to antibiotics in the future.

#### 2.7.4. Antimicrobials from Natural Sources

In addition, some antimicrobials from natural sources have also been used to prevent *V. alginolyticus* infection. The sources of this method are very rich. For example, compared with the parent phages, Phage Endolysins Lysqdvp001 has better lytic and antibacterial activity, which might act as possible antimicrobials against multidrug oppose *V. parahaemolyticus* and *V. alginolyticus* [100]. Moreover, Exogenous malic acid and taurine can improve the survival ratio of zebrafish infected with *V. alginolyticus* [101]. Additionally, supplementary feeding of astaxanthin has been proven to be resultful in consolidating fish immunocompetence, and illness resistance oppose *V. alginolyticus* infection [102]. Moreover, extracts from the secondary metabolites of some heterotrophic bacteria can also inhibit the growth of *V. alginolyticus* [103].

#### 2.7.5. Vaccines

- Attenuated live vaccines

There are many studies on live attenuated vaccines of *V. alginolyticus* (Table 7). The high vaccination potency makes the hfq mutant a prospective attenuated live vaccine to defend fish from pathogenic *V. alginolyticus* infection [104]. Moreover, ∆*acfA* can induce a resultful and long-lasting immune response in pearl gentian grouper and can also be a resultful attenuated live vaccine candidate for the prevention of *V. alginolyticus* infections [105]. In addition, a resultful live vaccine is the HY9901 ∆hop mutant, which can be used against *V. alginolyticus* in grouper [106]. Similarly, another resultful live attenuated vaccine against *V. alginolyticus* infection in pearl gentian fish is HY9901ΔvscB mutant [107]. Results of a study indicated ∆*clpP* showing great potential to be a live attenuated vaccine as well [108]. The previously mentioned attenuated candidate vaccine of *V. harveyi* has a cross-protective effect on *V. alginolyticus* [24], while the live attenuated vaccine of *V. alginolyticus* T3SS has also been proven to be protective [109]. A recent study has developed an unreported method of preparing live attenuated *V. alginolyticus* vaccination by incubating the bacteria in a high concentration of magnesium to attenuate the virulence of the bacteria [110].

- Recombinant vaccines

Recombinant vaccines have also been studied in *V. alginolyticus* prevention. Fish vaccinated with recombinant OmpU vaccine were proved to be highly resistant to attack by *V. alginolyticus*, and the OmpK and OmpW vaccines are similar to their principle [111,112]. Moreover, the FlaC protein demonstrated a significant immune protection function against *V. alginolyticus* infection [113], and they are expected to become potential vaccines against *V. alginolyticus*.

Since aquatic product diseases are often caused by multiple pathogens, it is particularly important to develop vaccines against more than one pathogen [114]. The two vaccines mentioned above in other *Vibrio* vaccines also have the same immune effects on *Vibrio alginolyticus* [16,27].

- DNA vaccines

A DNA vaccine is also a promising technology used against *V. alginolyticus*. It has been proved that the plasmid DNA coding flagellin *flaA* gene can effectively immunize red snappers. [115]. In another study, the *ompW* gene was inserted into the pcDNA plasmid to obtain a DNA vaccine. After the animal infection test, the RPS of this vaccine was 92.53% [116]. Additionally, among the 16 DNA vaccines constructed in an experiment, 3 (AT730_22220, AT730_22910 and AT730_21605) of them tended to be potential candidates for multivalent vaccines as being protective as opposed to *V. alginolyticus* infection with 47–66.7% added survival contrasted to the control [117].

- BGs vaccines

Recently, a newly constructed *V. alginolyticus* ghost, VaBGs, has been proven to be a secure and resultful vaccine for preventing *V. alginolyticus* infection. Moreover, this kind of BGs vaccine has also been developed in other pathogens as a new technology [118].

The research on the control technology of *V. alginolyticus* is also relatively mature, and the types of vaccines against *V. alginolyticus* are diverse. Turning these vaccines into more commercial vaccines for application is the next goal of *V. alginolyticus* Prevention.

**Table 7:** Control and prevention strategies of *V. alginolyticus.* NR: Not Relevant, None: the method has not been tested in vivo or relevant data has not been found.

Pathogen	Prevention and Control Technology	Concrete Measure/ Vaccine Type	Host	Vaccine Antigen Components	Route of Infection	Ref.
*V. alginolyticus*	Antibiotics	Florfenicol, Enrofloxacin, Terramycin	Nile tilapia	NR	Injection (IP)	[92]
		Doxycycline, Tetracycline	Hippocampus	NR	Injection (IP)	[12]
	Probiotics	*Pseudoalteromonas* sp. IBRL PD4.8	None	NR	None	[95]
	Antimicrobials from natural sources	Phage Endolysins Lysqdvp001	None	NR	None	[100]
		Exogenous malic acid, Taurine	Zebrafish	NR	Injection (IP)	[101]
		Astaxanthin	Fish	NR	Oral	[102]
		Secondary metabolite (From *Bacillus* sp. strain JS04)	None	NR	None	[103]
	Bacteriophages	Phage VP01, Phage VEN Phage HH109, Phage valsw3-3	None	NR	None	[96,97,98,99]
	Vaccines	Inactivated	*E. coioides*	VICV	Injection (IP)	[16]
		Attenuated	Zebrafish	*hfq* mutant	Injection (IM)	[104]
			Pearl gentian grouper	∆*acfA*, HY9901∆*hop*, HY9901Δ*vscB*, ∆*clpP* mutant	Injection (IP)	[105,106,107,108]
			Japanese flounder	Attenuated mutant *V. harveyi* T4DM	Injection (IP), Bath	[24]
			Zebrafish	Attenuated *V. alginolyticus* T3SS	Injection (IM)	[109]
			Zebrafish	Attenuated *V. alginolyticus* (Mg)	Injection (IM)	[110]
		Recombinant	Juvenile sea bass	Expressed r-OmpK of *Vibrio*	Injection (IP)	[27]
		DNA	Red snapper	Plasmid encoding *flaA*	Injection	[115]
			Crimson snapper	pcDNA plasmid with OmpW	Injection (IM)	[116]
		BGs	Large yellow croaker	*V. alginolyticus* bacterial ghosts	Injection (IP)	[118]

## 3. Discussion

Aquaculture production in Asia has increased significantly in recent decades, while worryingly, the development speed of aquatic disease prevention and control technology has been far behind the development of this fast-paced industry.

Vibriosis will remain to be one of the major challenges for the development of Asian aquaculture, with increasing breeding density and shorter breeding cycles. As one of the emerging methods to prevent and control vibriosis in aquaculture, the vaccine has a good application prospect. It is foreseeable that the future development trend of the *Vibrio* vaccine will be more convenient, cost-effective and environmentally friendly [14].

However, the application of *Vibrio* vaccines in Asian aquaculture still faces the following problems currently. First and foremost, through our investigation, we found that most of the current *Vibrio* vaccines are injected and rarely bathed. In aquaculture, fish often have a complex growth environment and a large number, so the implementation of injectable vaccines in the actual production is difficult. Moreover, the protective effect of oral vaccines is limited [119]. Secondly, the safety of fish vaccines has always been a concern [120]. It is truly that most of the *Vibrio* vaccines we investigated are still in the lab testing phase, which is either immature or subject to regulatory restrictions due to their uncertain security. Last but not least, the aquaculture industry chain and regulatory system in many Asian countries have yet to be perfect. These are important because only positive interaction between industry participants gives incentives to innovate and development of new technologies which could solve the facing problems of aquaculture industry.

The development of *Vibrio* vaccines in Asian aquaculture is still in the innovative research phase, and most innovations are progressive. However, there is no doubt that once the vaccine technology is mature, it will be widely used as the most cost-effective method of combating *Vibrio* diseases. Meanwhile, the aquaculture industry is exploring other solutions for *Vibrio* diseases as well, such as bacteriophages, nanoparticles and breeding for disease resistance.

## 4. Conclusions

In this review, we introduce and discuss the control and prevention strategies of seven species of *Vibrio* in Asian fish culture. Great progress has been made in this field in the past two decades. Although antibiotic treatment is widely used, with the gradual increase of *Vibrio* resistance to antibiotics, it is urgent to find some alternative non-chemical prevention and treatment methods. Probiotics, bacteriophages, natural extracts and other methods are created to meet the requirements of environmental protection and sustainable development in Asian fish culture and have great development potential in the future. There are more and more types of vaccines against *Vibrio:* attenuated, inactivated, recombinant and DNA vaccines. Even now, the *Vibrio* mRNA vaccine applied in aquaculture is being studied. The effectiveness of most of these vaccines has been verified in the laboratory.

When we investigated the *Vibrio* vaccine technology, we found that most of the vaccine trials were still limited to laboratory investigation, and its use in the actual aquaculture environment still needs further testing. *Vibrio* vaccine serves the aquaculture industry, so designers should also fully consider the development cost of the vaccine and the vaccination cost in practical application when developing it, which is quite an important factor in future vaccine promotion.

The seven *Vibrios* were selected in this paper because they have a related vaccine or the vaccine created is in urgent need. When investigating the prevention and control strategies of *Vibrio*, we found that at least 30 kinds of *Vibrios* can cause harm to the Asian fish culture industry, but less than 10 kinds of *Vibrios* have vaccine prevention and control technology. The *V. cholerae* selected in this paper is one of the *Vibrios* that has not yet found vaccine control technology. It can be seen that there is still a big gap in the prevention and control strategy of *the Vibrio* vaccine in Asian fish culture.

In general, this paper summarizes several existing measures and the latest technology of *Vibrio* control, which will provide ideas for better prevention and control of vibriosis in future Asian fish cultures.

## Figures and Tables

**Table 2:** Control and prevention strategies of *V. vulnificus.* NR: Not Relevant.

Pathogen	Prevention and Control Technology	Concrete Measure/ Vaccine Type	Host	Vaccine Antigen Components	Route of Infection	Ref.
*V. vulnificus*	Vaccines	Inactivated	Tilapia (Sex reversed hybrid)	Atypical *V. vulnificus* (Formalin killed cells)	Injection (IP)	[34]
			*E. coioides*	Vh + Vv + Va inactive vaccine and ISKNV whole cell inactive vaccine	Injection (IP)	[16]
		Subunit	Japanese eel	Expressed OmpU of *V. vulnificus*	Injection (IP)	[35]
		Multivalent	Japanese eel	Recombinant Omp containing both OmpA and OmpU	Injection (IP)	[36]
			European eel	Trivalent outer membrane protein (OmpⅡ-U-A)	Injection (IP)	[37]

**Table 3:** Control and prevention strategies of *V. parahaemolyticus.* NR: Not Relevant.

Pathogen	Prevention and Control Technology	Concrete Measure/ Vaccine Type	Host	Vaccine Antigen Components	Route of Infection	Ref.
*V. parahaemolyticus*	Vaccines	Inactivated	Tiger grouper	Vaksin polivalen *Vibrio* (formalin killed cells)	Injection, Bath	[43]
		Recombinant	Juvenile sea bass	Expressed r-OmpK of *Vibrio*	Injection (IP)	[27]

**Table 4:** Control and prevention strategies of *V. cholerae.* NR: Not Relevant, None: the method has not been tested in vivo or relevant data has not been found.

Pathogen	Prevention and Control Technology	Concrete Measure/ Vaccine Type	Host	Route of Infection	Ref.
*V. cholerae*	Antibiotics	Neomycin, Doxycycline	Bluegill sunfish	Injection (IP)	[49]
	Edible antibody	Anti-non-O1 *V. cholerae* egg yolk powder	Carp	Injection	[51]
	Vaccines	None	None	None	None

**Table 6:** Control and prevention strategies of *V. mimicus.* NR: Not Relevant.

Pathogen	Prevention and Control Technology	Concrete Measure/ Vaccine Type	Host	Vaccine Antigen Components	Route of Infection	Ref.
*V. mimicus*	Antibiotics	Florfenicol, Gentamicin, Lomefloxacin	Southern catfish	NR	Injection (IP)	[83]
	Vaccines	DNA	Grass carp	BGS and ICLP	Injection (IP), Oral	[84]
		Attenuated	Yellow catfish	*V. mimicus* SCCF01 (attenuated)	Bath	[89]

## Data Availability

Not applicable.
